# SRC and MEK Co-inhibition Synergistically Enhances the Anti-tumor Effect in Both Non-small-cell Lung Cancer (NSCLC) and Erlotinib-Resistant NSCLC

**DOI:** 10.3389/fonc.2019.00586

**Published:** 2019-07-30

**Authors:** Man Yuan, Lin-feng Xu, Juan Zhang, Si-yuan Kong, Man Wu, Yuan-zhi Lao, Hua Zhou, Li Zhang, Hongxi Xu

**Affiliations:** ^1^School of Pharmacy, Shanghai University of Traditional Chinese Medicine, Shanghai, China; ^2^Shanghai Chempartner Co., Ltd, Shanghai, China; ^3^Institute of Cardiovascular Disease of Integrated Traditional Chinese Medicine and Western Medicine, Shuguang Hospital, Shanghai University of Traditional Chinese Medicine, Shanghai, China

**Keywords:** non-small-cell lung cancer, MEK and SRC inhibitor, trametinib, bosutinib, synergistic effect

## Abstract

Non-small-cell lung cancer (NSCLC) is the predominant form of lung cancer, and it is regulated by a complex signal transduction network. Single-agent targeted therapy often results in acquired resistance, which leads to treatment failure. In this study, we demonstrated that a combination of the kinase inhibitors trametinib and bosutinib can synergistically suppress the growth of NSCLC by inhibiting both the mitogen-activated protein kinase (MAPK) and proto-oncogene tyrosine-protein kinase (SRC) pathways. The combination was profiled against a panel of 22 NSCLC cell lines, including one erlotinib-resistant cell line, and this combination was found to show synergistic effects against 16 cell lines. NSCLC cell lines (HCC827, HCC827-erlotinib-resistant, and H1650) were treated with trametinib, bosutinib, or a combination of these drugs. The drug combination inhibited colony formation and induced cell apoptosis. A mechanism study showed that the phosphorylation of multiple kinases in the epidermal growth factor receptor (EGFR) signaling pathway in NSCLC was down-regulated. In addition, the combination significantly attenuated tumor growth of HCC827 xenografts with low toxicity. Our findings provide a theoretical basis for further study of the combination of MAPK and SRC pathway inhibitors in NSCLC, especially in the treatment of erlotinib-resistant NSCLC.

## Introduction

Lung cancer is the most common type of cancer that continues to be the leading cause of cancer deaths worldwide (1–4). Non-small-cell lung cancer (NSCLC), which accounts for approximately 85% of all lung cancer patients, can be further subclassified into squamous cell carcinoma, large-cell carcinoma, and adenocarcinoma (5). Surgery, radiotherapy and chemotherapy are the most common treatments for lung cancer. However, the current outcome of treatment is unsatisfactory, with only 15% 5-year survival (2, 3). The poor outcome reflects the advanced disease stage and degree of metastasis at diagnosis, as well as the fact that most patients develop resistance to the treatment given and quickly experience progression of their disease.

Epidermal growth factor receptor (EGFR) is a member of the ErbB family of receptors. It is closely related to cell proliferation, apoptosis, and epithelial mesenchymal transformation (EMT). EGFR is a well-characterized mutated oncogene in NSCLC, and its mutations mainly include an exon 21 L858R point mutation and an exon 19 deletion (19 del) mutation (2). EGFR mutations are widely involved in tumor proliferation and survival, and are also identified as the therapeutic target of EGFR tyrosine kinase inhibitors (TKIs). Gefitinib and erlotinib, the first-generation EGFR-TKIs, have become the standard chemotherapy for advanced NSCLC with activating mutations in EGFR (6–8). It is reported that patients with sensitive EGFR experience a pronounced initial effect of this treatment, achieving a median progression-free survival (PFS) of 8–16 months. However, despite these dramatic responses, acquired resistance (9, 10) to EGFR-TKIs develops in almost all patients, usually within 1 year, thus limiting improvement in patient outcomes.

Therefore, research has begun to apply targeted drug combinations to abrogate signaling pathway cross-talk and restore treatment sensitivity (11–13). Research has found that a combination of mitogen-activated protein kinase kinase (MEK) and proto-oncogene tyrosine-protein kinase (SRC) inhibition suppresses melanoma cell growth and invasion (14). Studies have also found that combined treatment using targeted MEK and SRC inhibitors synergistically abrogates tumor cell growth and induces mesenchymal–epithelial transition in non-small-cell lung carcinoma (5). It was reported that the Src inhibitor dasatinib with the MEK inhibitor selumetinib is an effective combination in Ras-mutant erlotinib-resistant models (15). These research findings indicate that Src and MEK inhibitors display synergistic effects, which may therefore offer a better therapeutic advantage.

Through a screening assay of cell line toxicity on a panel of lung cancer cells, we found that trametinib and bosutinib, inhibitors of MEK and SRC kinases, respectively, showed potential synergistic inhibition effects on cell proliferation in both NSCLC and erlotinib-resistant NSCLC. In this study, we investigate the possible synergistic role of trametinib and bosutinib in NSCLC therapy. A variety of *in vitro/in vivo* assays were used to clarify the molecular targets of the co-treatment.

## Materials and Methods

### Cell Culture and Chemicals

Human lung cancer cell lines obtained from the American Type Culture Collection (ATCC, Manassas, VA, USA) were cultured in RPMI 1640 medium supplemented with 10% fetal bovine serum (PAA, A15-101) and 10 U/ml penicillin–streptomycin (Gibco/Invitrogen, 15140-122) at 37°C in a humidified atmosphere with 5% CO_2_. Erlotinib (S7786), bosutinib (S1014), and trametinib (S2673) were purchased from Selleck China.

### Generation of Erlotinib-Resistant HCC827 Lung Cancer Cell Line

The erlotinib-resistant HCC827 lung cancer cell line was generated as described previously (16). Briefly, the HCC827 cells were cultured in growth medium in the presence of vehicle (0.1% DMSO) or gradually increasing concentrations of erlotinib to result in the resistant cell line that was designated HCC827-ER.

### MTT Assay and Determination of IC_50_

Cell proliferation was detected as described before (17). Briefly, cell line was seeded into a 96-well plate; after attachment, the cells were then treated with different doses of trametinib or bosutinib, alone or in combination for 24 h. After treatment, 10 μl of 5 mg/ml 3-(4,5-dimethylthiazol-2-yl) 2,5-diphenyltetrazolium bromide (MTT) solution was added to each well and incubated with the cells for 3 h. After removing the medium, 100 μl of DMSO was added and then the absorbance at 570 nm was measured. The cell viability was normalized and is presented as a percentage of the control.

The interaction effect between bosutinib and trametinib was analyzed using the combination index (CI) (18, 19). The CI value is defined by the Loewe Additivity equation: CI = (D)1/(Dx)1 + (D)2/(Dx)2, where (Dx)1 and (Dx)2 are the concentrations for D1 (bosutinib) and D2 (trametinib) alone that inhibit x% cell growth, and (D)1 and (D)2 are the concentrations of bosutinib and trametinib in combination that result in identical cell growth inhibition. Each CI value is the mean value of three independent experiments.

### Colony Formation Assay

Cells were seeded at specific cell densities in six-well plates (1 × 10^5^, 2 × 10^5^, and 1.5 × 10^5^ for H1650, HCC827, and HCC827 ER cells, respectively), and the plates were incubated overnight. The cells were then treated with different doses of trametinib or bosutinib, alone or in combination, for the indicated periods. Cells were washed with PBS and then cultured in fresh medium for 14 days. Subsequently, the cells were fixed and stained with 0.1% crystal violet. Colonies of at least 50 cells observed under a microscope were counted as one positive colony.

### Annexin V-FITC/PI Staining Assay

After treatment, the cells were harvested, washed twice with ice-cold PBS, and then stained using an Annexin V-FITC/PI Cell Apoptosis Detection Kit (BD Biosciences) according to the manufacturer's instructions. In brief, 1 × 10^6^ cells were resuspended in 400 μl of binding buffer, and 5 μl of 2 mg/ml Annexin V and 5 μl of 20 μg/ml PI were then added. After 15-min incubation in the dark, the stained cells were quantified by a FACScan flow cytometer, and the data were analyzed using FACSCalibur software. The cells in the early stage of apoptosis were Annexin V positive and PI negative, whereas the cells in the late stage of apoptosis were both Annexin V and PI positive.

### Western Blotting Analysis

The equivalent amounts of protein were fractionated using 10% SDS–PAGE, and then the proteins were transferred to a polyvinylidene difluoride membrane. The membranes were blocked with 0.1% TBS/T (0.1%) containing 5% non-fat milk. After blocking for 1 h, membranes were incubated with different antibodies diluted in 3% bovine serum albumin in washing buffer. The following primary antibodies were used in this study: Y419-phospho-SRC (Abcam#ab185617), Y529-phospho-SRC (Abcam#ab4817), SRC (Abcam#ab109381), S235/236-phospho-S6 (CST#4856), S240/244-phospho-S6 (CST#5364), S6 (CST#2217), Y1068-phospho-EGFR (CST#2236), Y854-phospho-EGFR (CST#6963), EGFR (CST#2236), p44/42 (CST#9107), phospho-p44/42 (CST#4370), AKT (CST#9272), and phospho-AKT (CST#9271). Unless indicated otherwise, all antibodies were used at a 1:1,000 dilution.

Afterward, the membrane was washed and probed with the HRP-conjugated secondary antibodies at room temperature for 1 h. The protein bands were detected using an ECL kit (Pierce, Rockford, IL, USA).

### *In vivo* Animal Experiment

This study was carried out in accordance with the recommendations of the Guidelines for the Care and Use of Laboratory Animals, and the protocols were approved by the Institutional Animal Care and Use Committee of Shanghai University of Traditional Chinese Medicine. Specific pathogen-free BALB/c male nude mice (4 weeks old) were purchased from the Experimental Animal Center of the Chinese Academy of Science (Shanghai, China). HCC827 cells (5 × 10^6^ cells per mouse) were re-suspended in 200 μl of Matrigel (BD, USA) and then were subcutaneously inoculated into the dorsal flank of nude mice. After tumors had established (~75 mm^3^), the mice were randomly divided into four groups (six mice per group) for intragastric administration with either vehicle control (1% Tween−80 in saline) (Group 1), trametinib at 0.4 mg/kg every day for 2 weeks (Group 2), bosutinib at 60 mg/kg every day for 2 weeks (Group 3), or a combination of trametinib and bosutinib. The body weight and tumor sizes of all mice were recorded every 2 days. The length and width of the tumor were measured by a digital caliper, and the tumor volumes were calculated according to the formula: [(shortest diameter)^2^ × (longest diameter)]/2. After 14 days of treatment, the mice were humanely killed, and their tumors were resected, weighed, and photographed.

### Statistical Analyses

Data are presented as means ± SD from three independent experiments. Student's two-tailed *t-*test was used for comparison between two different groups. ANOVA was used for multiple comparisons. All *P*-values < 0.05 were considered to be statistically significant.

## Results

### Bosutinib and Trametinib Inhibited Cell Proliferation Synergistically in Lung Cancer Cell Lines

**Figure 1:**
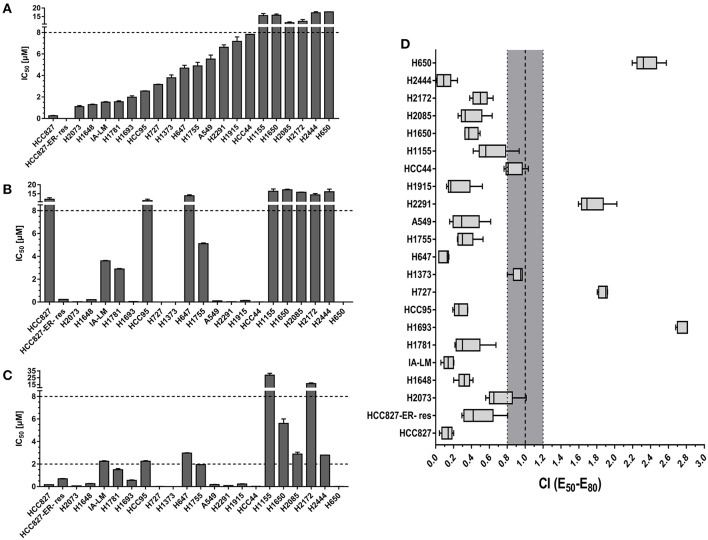
The combination of MEK inhibitor trametinib with SRC inhibitor bosutinib promoted synergistic inhibition of cell growth in NSCLC cell lines. Cell proliferation IC_50_ plots (mean ± SD) for a panel of 22 NSCLC cell lines treated with bosutinib **(A)**, trametinib **(B)**, or the combination of bosutinib and trametinib **(C)** for 24 h. Data were tabulated from three independent experiment sets. **(D)** Combination index (CI) box plots of bosutinib and trametinib co-treatment at a ratio of 1:1 on the cell line panel. Combination index of CI < 0.8 indicates synergism, CI from 0.8 to 1.2 indicates additive effect, and CI > 1.2 indicates antagonism.

The proliferation inhibition effects of bosutinib and trametinib as single drugs and as a combination on a collection of 22 lung cancer cell lines were evaluated. For single drug treatments, the results indicated that trametinib showed overall a better inhibition effect than bosutinib. After treatment, 11 out of 22 cell lines (50%) were observed to be sensitive to trametinib (cell proliferation IC_50_ < 2 μM), while 6 cell lines (27%) were considered to be resistant to it (cell proliferation IC_50_ > 10 μM) (Figure 1A). For bosutinib single-drug treatment, we found that only six cell lines (27%) were sensitive. The percentage of resistant cell lines was also 27% (Figure 1B). Next, the effect of the combination of bosutinib and trametinib (1:1 molar ratio) on the proliferation of these 22 cell lines was investigated (Figure 1C). The drug combination indices (CI) for the 50–80% growth inhibition ranges were then calculated using the Loewe Additivity model (Figure 1D). We found that 19 of the cell lines showed synergistic effects with CI values < 1. Among these cell lines, the HCC827 cell line showed the strongest synergistic effect (CI valve: 0.14), and this effect was also observed in the erlotinib-resistant HCC827 (HCC827ER) cell line. The synergistic effect of these two compounds was further studied on both HCC827 and HCC827ER cells. Another cell line with an EGFR mutation, the H1650 cell line, was also used in our study later.

### Bosutinib and Trametinib Combination Suppressed Cell and Colony Growth in Three NSCLC Lines

**Figure 2:**
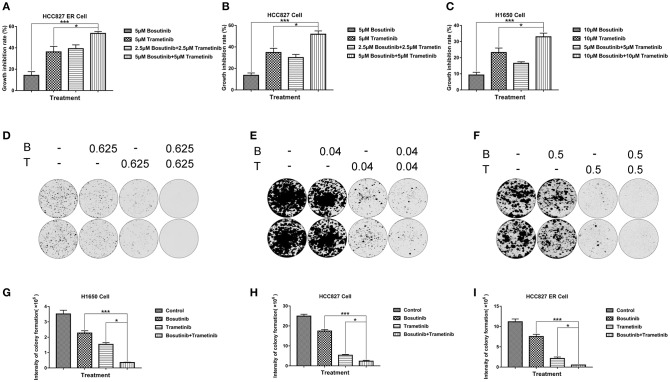
The combination of trametinib with bosutinib synergistically reduced the viability of NSCLC *in vitro*. **(A)** H1650, **(B)** HCC827, and **(C)** HCC827 ER cell lines were seeded in 96-well plates. Trametinib, bosutinib, or their combination was added for 24 h. Cell viability was determined using the MTT assay. **(D)** H1650, **(E)** HCC827, and **(F)** HCC827 ER cell lines were seeded at specific cell densities in six-well plates and were incubated overnight. The cells were treated with different concentrations of trametinib, bosutinib, or their combination for 24 h. Then, the media were replaced with complete medium without drugs, and the cells were cultured for 14 days. Histograms of quantitative analysis of the colony formation assay: **(G)** H1650, **(H)** HCC827, and **(I)** HCC827ER cell lines. The data are presented as mean ± S.D. ^*^P < 0.05, ^***^P < 0.001.

Based on the screening results of the 22 lung cancer cell lines, the cytotoxic effects of the two drugs and their combinations were further confirmed in three NSCLC lines (HCC827, HCC827ER, and H1650) using the MTT assay after treatment for 24 h. Based on the IC_50_ value of these two compounds on the three cell lines, here the dosage we used for H1650 was 10 μM, while the dosage for both HCC827 and HCC827ER was 5 μM. As shown in Figures 2A–C, the inhibition percentage for single treatment of bosutinib was around 10–20% in all three cell lines, while the cells were more sensitive to trametinib, for which the rate was around 30 to 40%. As compared with single treatment, the combination of the drugs greatly increased the cell growth inhibition rate. This rate reached nearly 60% in both HCC827 and HCC827ER cells, and similar results were observed in H1650 cells.

In order to study the potential effect on long-term proliferation, a colony formation assay was used in our study. Cells were seeded and treated with different concentrations of bosutinib and trametinib, or their combination for 24 h. The clonogenic assays showed that bosutinib and trametinib had a synergetic effect on colony formation on both HCC827 and HCC827 ER cells (Figures 2D–I). Interestingly, a combination of 0.625 μM bosutinib and 0.625 μM trametinib completely blocked colony formation in HCC827 ER cells, while either agent alone showed no such effect. The synergistic inhibitory effect of these two drugs was also observed in H1650 cells (Figures 2F–I). Colony formation was greatly inhibited after treatment with 0.5 μM of bosutinib and trametinib for 24 h, and was only slightly inhibited by either agent alone.

### Bosutinib and Trametinib Induce Apoptosis Synergistically on NSCLC and Erlotinib-Resistant NSCLC

**Figure 3:**
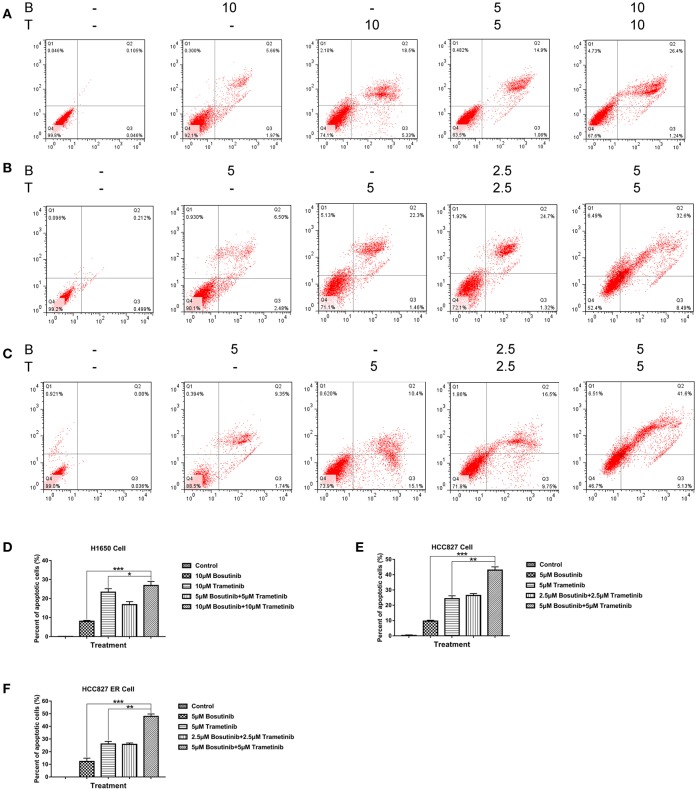
Trametinib and bosutinib trigger apoptosis in NSCLC cell lines synergistically. Annexin V/PI flow cytometric analysis of drug-treated **(A)** H1650, **(B)** HCC827, and **(C)** HCC827 ER cells. Cells treated with trametinib, bosutinib, or their combination for 24 h. The cells were then collected and were double-stained with an FITC-conjugated anti-Annexin V antibody and PI. The analyses were performed using a flow cytometer. Histograms of quantitative analysis of colony formation assay. **(D)** H1650, **(E)** HCC827, and **(F)** HCC827ER cell lines. The data are presented as mean ± S.D. ^*^P < 0.05, ^**^P < 0.01, ^***^P < 0.001.

To investigate the effect of bosutinib and trametinib on NSCLC, an apoptosis assay was performed using flow cytometric analysis. The cells were treated with different concentrations of bosutinib and trametinib, or their combination. After treatment for 24 h, cells were harvested and stained with Annexin V/PI for analysis. As shown in Figure 2, the number of apoptotic cells in both the HCC827 and HCC827 ER cells was slightly increased by treatment with bosutinib and trametinib alone, whereas combined treatment with bosutinib and trametinib increased the percentage of apoptotic cells to 32.6% in HCC827 and 41.6% HCC827 ER cells (Figures 3B,C). Our results showed that when the two drugs were used together, half of the dosage could give a promising result (Figures 3D–F). Similar results were also observed in H1650 cells (Figure 3A). Additionally, the apoptotic effect of the drug was further confirmed by Western blotting analysis (Supplementary Figure 1). The activities of caspase-dependent pathway markers, including caspase-3, caspase-9, and PARP, were investigated. The activity of caspases-3 and-9 in the combined treatment cells was significantly higher than that in the single treatment cells, since they contained less mount of pro-caspases-3 and-9. As a result of the activation of the caspase-signaling pathway, the amount of cleaved PARP was significantly increased in the combined treatment cells. Consistent with the results of flow cytometric analysis, the combination of bosutinib and trametinib also showed synergistic effects in Western blot analysis.

### Bosutinib and Trametinib Inhibited Cell Growth on NSCLC and Erlotinib-Resistant NSCLC by Synergistically Targeting the EGFR Signaling Pathway

**Figure 4:**
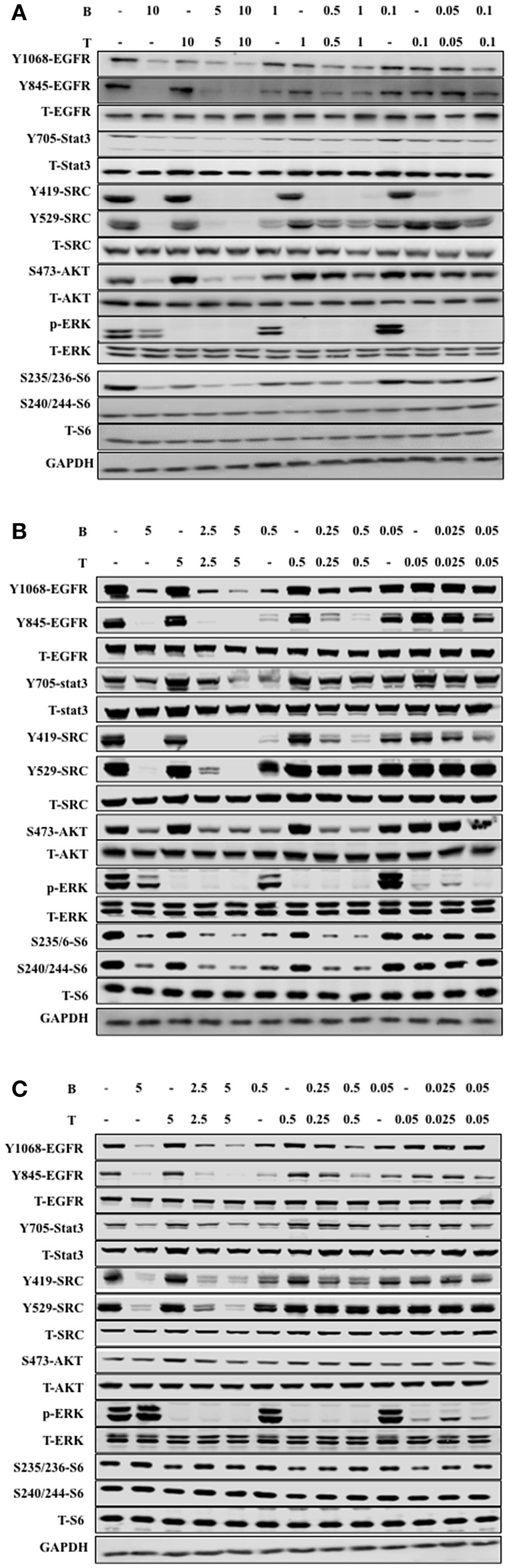
The combination of trametinib and bosutinib enhanced anti-tumor activity through EGFR *in vitro*. **(A)** H1650 cells, **(B)** HCC827 cells, and **(C)** HCC827ER cells were treated with various concentrations of trametinib, bosutinib, or their combination for 24 h. The cells were harvested, and the effects of the drugs on the protein expression levels related to the EGFR signaling pathway were detected by Western blot analysis (*n* = 3 for all experiments).

We further studied the mechanism of combined bosutinib and trametinib on H1650, HCC827, and HCC827ER cell lines. Since bosutinib and trametinib are inhibitors of SRC and MEK, respectively, the phosphorylation and total protein levels of SRC and its downstream signals STAT3, AKT, ERK, and S6 were analyzed after bosutinib, trametinib, or combination treatment. As shown in Figure 4 and Supplementary Figure 2, bosutinib treatment dramatically decreased the phosphorylation level of SRC and the downstream phosphorylated proteins, such as STAT3, AKT, and S6 in all three cell lines, while trametinib specifically inhibited the expression level of phosphorylated ERK. Co-treatment with these two drugs synergistically decreased the phosphorylation levels of SRC Y419 and S6.

It is well-known that Src can phosphorylate EGFR on Y845, while Y1068 is one of the major autophosphorylation sites of EGFR, so we studied the effect of combined bosutinib and trametinib on these two sites. The results indicated that bosutinib treatment significantly decreased the phosphorylation level of EGFR on both Y845 and Y1068, and this effect was intensified by co-treatment with trametinib in these three cell lines. Specifically, we carefully compared the effects of the co-treatments on phosphorylation of EGFR in the HCC827 and HCC827ER cell lines. Our study showed that HCC827ER seemed to be more sensitive than HCC827 cells to co-treatment. As shown in Figures 4B,C, a combination of bosutinib and trametinib showed synergistic effects on down-regulation of EGFR Y1068 at both higher (5 μM) and lower (0.5 μM) dosages, while this effect was seen only at higher dosage in HCC827 cells.

### The Combination of Bosutinib and Trametinib Improved the Inhibitory Effect in HCC827 Cell Xenografts in Nude Mice

**Figure 5:**
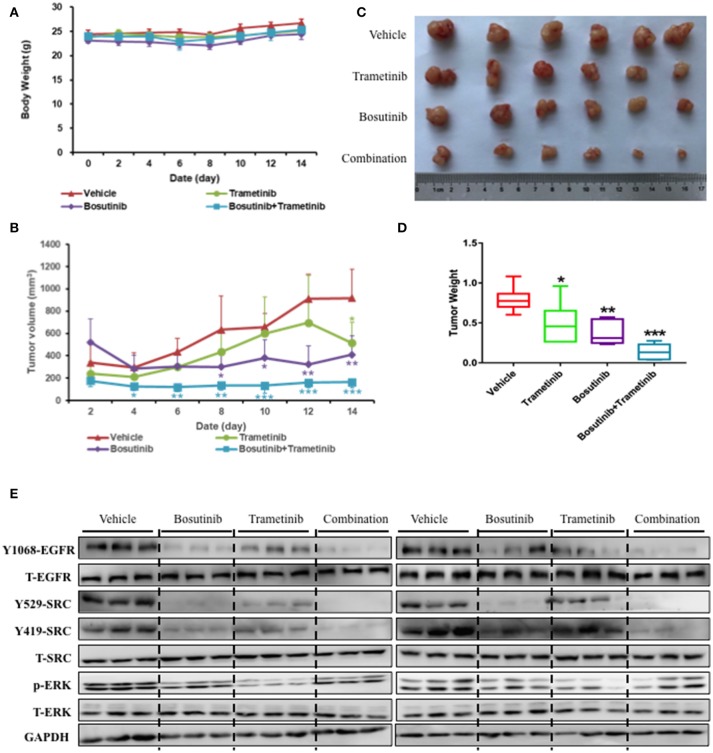
The combination of trametinib and bosutinib showed synergistic effects against xenografted tumors in nude mouse. HCC827 cells (1 × 10^6^ cells per mouse) were implanted subcutaneously into the dorsal flank of nude mice. When the tumors reached approximately 75 mm^3^, the tumor-bearing mice were then treated with vehicle, trametinib (e.g., 0.4 mg/kg), bosutinib (e.g., 60 mg/kg), or their combination once daily for 14 days. After treatment, the mice were killed. **(A)** Body weights and **(B)** tumor volumes of the animals were measured every 2 days. Tumors were removed, photographed **(C)**, and weighed **(D)**. **(E)** Western blotting analysis of the expression levels related to EGFR signaling pathway in mouse tumor sections. Data are expressed as means ± SD. ^*^P < 0.05, ^**^P < 0.01, ^***^P < 0.001 compared to the control (*n* = 6).

To further investigate the inhibition effect of bosutinib and trametinib on NSCLC growth and development, HCC827 cells were injected subcutaneously into the dorsal flank of nude mice. Bosutinib (60.0 mg/kg, p.o.), trametinib (0.4 mg/kg, p.o.), or a combination of these was administered every day for 14 days. The mice were treated for 14 days in our study, since the tumor volume was around 1,000 mm^3^ in the control group, and it is thought unethical if the tumor volume exceeds 1,000 mm^3^. The result showed that there was no significant difference in body weight between the vehicle and the three drug-treatment groups (Figure 5A). Treatment with bosutinib and trametinib did not alter cell morphology, nor did it cause bleeding in the heart, lung, kidney, or liver (Supplementary Figure 3). Compared with the vehicle control group, the combination of bosutinib and trametinib could significantly reduce the tumor volume of the mice, as seen on day 14. The mean tumor volumes measured on day 14 for the vehicle control, bosutinib, and trametinib groups were 916 ± 260, 513 ± 185, and 408 ± 167 mm^3^, respectively (Figure 5B). The tumor inhibition effect of the combination was significantly stronger than single treatment with bosutinib and trametinib. As shown in Figure 5, the tumor volume was dramatically decreased to 163 ± 185 mm^3^ after treatment for 14 days. A similar inhibitory effect was observed in the growth of tumors (Figure 5C). The results demonstrated that the tumor weight for mice treated with the combination of the two drugs was reduced by 82% compared with the control group, while the decreases with bosutinib and trametinib were 38 and 53%, respectively (Figure 5D).

To better understand the molecular mechanism of bosutinib and trametinib action on tumorigenesis *in vivo*, the phosphorylation levels of related proteins in tumor tissue were detected using Western blot analysis. As shown in Figure 5E and Supplementary Figure 4, the expression level of phosphorylated ERK was obviously inhibited by trametinib treatment, while the bosutinib administration down-regulated the level of phosphorylated EGFR and SRC as compared with the vehicle group. This result was reasonable, since bosutinib and trametinib are inhibitors of SRC and MEK, respectively. The combination treatment showed better effects on inhibition of these proteins in tumor tissue. The expression levels of the proteins in the co-treatment group were decreased significantly as compared with the single treatment group to the point of being hard to detect by Western blot. These results further supported our conclusion that the combination of bosutinib and trametinib showed better effects in a xenograft model as compared with single treatment.

## Discussion

SRC, a member of the non-receptor tyrosine kinase family, is involved in intracellular signaling pathways, which are usually associated with cell migration, proliferation, and apoptosis (20–22). Activation of SRC kinase is known to be associated with malignancy in a variety of human cancer types (23, 24), including NSCLC (25). SRC inhibitors are considered to be promising agents for NSCLC, but so far, disappointing results from clinical trials have delayed their clinical development (26–29). Bosutinib (Bosulif, SKI-606) is a second-generation TKI that specifically inhibits the kinase activity of Src (30, 31). Our experiments also confirmed that bosutinib treatment alone only resulted in modest anti-tumor effects in three NSCLC cells and xenografts. Regarding the mechanism, it is well-known that SRC kinase can activate EGFR by phosphorylating tyrosine 845 (Y845), which may contribute to complete receptor activation (32). In our study, the effect of bosutinib on phosphorylation of EGFR on Tyr845 was investigated. We found that bosutinib decreased the level of Y845 phosphorylation. Since the phosphorylation of EGFR on Y845 does not affect EGFR autokinase activity (32), the effect of bosutinib on phosphorylation of EGFR on Tyr1068, one of the major autophosphorylation sites, was also investigated. We found that the compound showed effects on this phosphorylation site. Interestingly, when bosutinib was combined with trametinib, the inhibitory effects were greatly increased both *in vivo* and *in vitro*. The combination showed a synergistic effect on down-regulation of the phosphorylation levels of EGFR on Tyr845 and Tyr1068.

The MAP kinase pathway is composed of Ras, Raf, MEK, and ERK kinases, which are essential for cell proliferation, inhibition of apoptosis, and migration. It plays a key role in tumorigenesis and growth of transformed cells. Blocking the mitogen-activated protein kinase (MAPK) pathway through MEK inhibitors has become a popular approach in cancer therapy (33). Trametinib (GSK1120212) is a MEK1/2 inhibitor with a longer half-life than previous MEK inhibitors (34, 35). Trametinib is approved by the FDA as a single drug or combined with dabrafenib for the treatment of incurable BRAF mutant melanoma (36, 37). Therapeutic strategies targeting MEK in NSCLC were also investigated, and previous studies indicated that trametinib showed promising results in phase 2 trials in NSCLC. Current studies mostly focus on trametinib treatment alone or in combination with chemotherapy in KRAS mutant NSCLC. This is because KRAS mutations are detected in 25% of lung adenocarcinomas (38–40), which are associated with reduced survival in NSCLC patients, as well as resistance to EGFR TKIs (39, 41–47). Studies found that trametinib has a strong activity in cases of BRAF mutation and its combination with dabrafenib has demonstrated substantial clinical activity in patients with the BRAF V600E mutant. That combination is now a standard treatment in the first-line setting (48, 49), although adverse events need to be managed (50). Here, we have studied, for the first time, the combination of trametinib and bosutinib in EGFR-mutant NSCLC, which is a well-characterized and commonly observed type of NSCLC. Our results showed that trametinib and bosutinib alone or in combination could inhibit NSCLC growth both *in vitro* and *in vivo*. The combination showed synergistic effects *in vitro*, which also enhanced the inhibitory effect in xenograft models. The possible mechanisms for this may include the fact that the combination inhibited counteracting signaling events, such as activation of PI3-kinase signaling as a direct response to MEK inhibition (51, 52). As shown in our results, the level of phosphorylated AKT was also up-regulated after trametinib treatment, but this was not observed in the combination group. Our results suggested that the combination of trametinib and bosutinib could be considered in future designs as rational approaches in EGFR-mutant NSCLC therapy.

Drug resistance is a major obstacle in the clinical treatment of NSCLC. Molecular mechanisms underlying acquired resistance to EGFR-TKIs in EGFR-mutated lung cancers have been studied. The secondary T790M mutation and the “oncogene kinase switch” are considered to be two major mechanisms of resistance. Research has indicated that a secondary T790M mutation is present in 50% of TKI-resistant patients with EGFR mutations, while other secondary resistance mutations (D761Y, L747S, and T854A) seem to be rare (53). Drug combinations have been proven to be effective in the treatment of erlotinib-resistant cells. It was reported that cetuximab plus saracatinib, which are Src and EGFR inhibitors, respectively, worked as an effective combination in T790M EGFR erlotinib-resistant cells. Co-treatment with SRC and MEK inhibitors showed effects in Ras mutant erlotinib-resistant models (15). In our study, the HCC827ER cell line was generated by gradually increasing the concentrations of erlotinib as described previously (16), which mimicked the situation in clinical practice when drug resistance develops after several months of treatment with the EGFR inhibitor erlotinib. The characteristics of this HCC827ER cell line were well-analyzed, as our previous study showed that the HCC827ER cells had a higher phosphorylation of AKT, ERK, Stat3, and S6, which were not attenuated by erlotinib treatment. No T790M or L858R mutation in the EGFR gene was found. Our results indicated that the combination of bosutinib and trametinib dramatically decreased the expression of S6, which may be one of the mechanisms for the synergistic effect in HCC827ER cells. However, the detailed mechanisms should still be investigated in further studies. Our results indicated that the combination may be a strategy for the treatment of erlotinib-resistant NSCLC without T790M or L858R mutation.

In conclusion, we have demonstrated that anti-cancer effects can be enhanced in both NSCLC lines and one erlotinib-resistant NSCLC line when bosutinib is combined with trametinib. These effects include reducing proliferation, inducing apoptosis, and down-regulating biomarker proteins. These data demonstrate that combining inhibition of the SRC and MAPK pathways should be considered in future designs in NSCLC and erlotinib-resistant NSCLC therapy.

## Data Availability

The raw data supporting the conclusions of this manuscript will be made available by the authors, without undue reservation, to any qualified researcher.

## Ethics Statement

This study was carried out in accordance with the recommendations of the Guidelines for the Care and Use of Laboratory Animals, and the protocols were approved by the Institutional Animal Care and Use Committee of Shanghai University of Traditional Chinese Medicine.

## Author Contributions

HX and MY conceived and designed the experiments. LZ, MW, and LX performed in vitro study. MY and SK performed the animal experiments. LZ, JZ, and LX analyzed the data. LZ, YL, and LX wrote the manuscript. HZ revised the manuscript.

### Conflict of Interest Statement

The authors declare that the research was conducted in the absence of any commercial or financial relationships that could be construed as a potential conflict of interest.

## Abbreviations

CI: combination index
EGFR: epidermal growth factor receptor
HCC827-ER: erlotinib-resistant HCC827 cell line
NSCLC: non-small-cell lung cancer.

## References

[1] Herbst RS Heymach JV Lippman SM. Lung cancer. N Engl J Med. (2008) 359:1367–80. 10.1056/NEJMra0802714 18815398 PMC10662965

[2] Siegel R Naishadham D Jemal A Cancer statistics 2012. CA Cancer J Clin. (2012) 62:10–29. 10.3322/caac.20138 22237781

[3] SEER Stat Fact Sheets Lung and Bronchus Cancer. National Cancer Institute (2015). Available online at: http://seer.cancer.gov/statfacts/html/lungb.html (accessed March 4, 2017).

[4] GLOBOCAN 2012: Estimated Cancer Incidence, Mortality and Prevalence Worldwide in 2012. World Health Organization (2015). Available online at: http://globocan.iarc.fr/Pages/fact_sheets_population.aspx (accessed March 4, 2017).

[5] Chua KN Kong LR Sim WJ Ng HC Ong WR Thiery JP. Combinatorial treatment using targeted MEK and SRC inhibitors synergistically abrogates tumor cell growth and induces mesenchymal–epithelial transition in non-small-cell lung carcinoma. Oncotarget. (2015) 6:29991–30005. 10.18632/oncotarget.5031 26358373 PMC4745777

[6] Rosell R Carcereny E Gervais R Vergnenegre A Massuti B Felip E. Erlotinib versus standard chemotherapy as first-line treatment for European patients with advanced EGFR mutation-positive non-small-cell lung cancer (EURTAC): a multicentre, open-label, randomised phase 3 trial. Lancet Oncol. (2012) 13:239–46. 10.1016/S1470-2045(11)70393-X 22285168

[7] Mok TS Wu YL Thongprasert S Yang CH Chu DT Saijo N. Gefitinib or carboplatin-paclitaxel in pulmonary adenocarcinoma. N Engl J Med. (2009) 361:947–57. 10.1056/NEJMoa0810699 19692680

[8] Sequist LV Yang JC Yamamoto N O'Byrne K Hirsh V Mok T Phase III study of afatinib or cisplatin plus pemetrexed in patients with metastatic lung adenocarcinoma with EGFR mutations. J Clin Oncol. (2013) 31:3327–34. 10.1200/JCO.2012.44.2806 23816960

[9] Suda K Mizuuchi H Maehara Y Mitsudomi T. Acquired resistance mechanisms to tyrosine kinase inhibitors in lung cancer with activating epidermal growth factor receptor mutation—diversity, ductility, and destiny. Cancer Metastasis Rev. (2012) 31:807–14. 10.1007/s10555-012-9391-7 22736441

[10] Yu HA Arcila ME Rekhtman N Sima CS Zakowski MF Pao W. Analysis of tumor specimens at the time of acquired resistance to EGFR-TKI therapy in 155 patients with EGFR-mutant lung cancers. Clin Cancer Res. (2013) 19:2240–7. 10.1158/1078-0432.CCR-12-2246 23470965 PMC3630270

[11] Sos ML Fischer S Ullrich R Peifer M Heuckmann JM Koker M. Identifying genotype-dependent efficacy of single and combined PI3K- and MAPK-pathway inhibition in cancer. Proc Natl Acad Sci USA. (2009) 106:18351–6. 10.1073/pnas.0907325106 19805051 PMC2757399

[12] Legrier ME Yang CP Yan HG Lopez-Barcons L Keller SM Perez-Soler R. Targeting protein translation in human non small cell lung cancer *via* combined MEK and mammalian target of rapamycin suppression. Cancer Res. (2007) 67:11300–8. 10.1158/0008-5472.CAN-07-0702 18056456

[13] Simpkins F Jang K Yoon H Hew KE Kim M Azzam DJ. Dual Src and MEK inhibition decreases ovarian cancer growth and targets tumor initiating stem-like cells. Clin Cancer Res. (2018) 24:4874–86. 10.1158/1078-0432.CCR-17-3697 29959144 PMC6557165

[14] Ferguson J Arozarena I Ehrhardt M Wellbrock C. Combination of MEK and SRC inhibition suppresses melanoma cell growth and invasion. Oncogene. (2013) 32:86–96. 10.1038/onc.2012.25 22310287 PMC3378628

[15] Formisano L D'Amato V Servetto A Brillante S Raimondo L Di Mauro C. Src inhibitors act through different mechanisms in non-small cell lung cancer models depending on EGFR and RAS mutational status. Oncotarget. (2015) 6:26090–103. 10.18632/oncotarget.4636 26325669 PMC4694888

[16] Xu L Meng X Xu N Fu W Tan H Zhang L. Gambogenic acid inhibits fibroblast growth factor receptor signaling pathway in erlotinib-resistant non-small-cell lung cancer and suppresses patient-derived xenograft growth. Cell Death Dis. (2018) 9:262. 10.1038/s41419-018-0314-6 29449529 PMC5833807

[17] Tian HL Yu T Xu NN Feng C Zhou LY Luo HW. A novel compound modified from tanshinone inhibits tumor growth *in vivo via* activation of the intrinsic apoptotic pathway. Cancer Lett. (2010) 297:18–30. 10.1016/j.canlet.2010.04.020 20494511

[18] Chou TC. Theoretical basis, experimental design, and computerized simulation of synergism and antagonism in drug combination studies. Pharmacol Rev. (2006) 58:621–81. 10.1124/pr.58.3.10 16968952

[19] Chou TC. Drug combination studies and their synergy quantification using the Chou-Talalay method. Cancer Res. (2010) 70:440–6. 10.1158/0008-5472.CAN-09-1947 20068163

[20] Chatzizacharias NA Kouraklis GP Giaginis CT Theocharis SE. Clinical significance of Src expression and activity in human neoplasia. Histol Histopathol. (2012) 27:677–92. 10.14670/HH-27.677 22473690

[21] Frame MC. Src in cancer: deregulation and consequences for cell behaviour. Biochim Biophys Acta. (2002) 1602:114–30. 10.1016/S0304-419X(02)00040-9 12020799

[22] Russello SV Shore SK. SRC in human carcinogenesis. Front Biosci. (2004) 9:139–44. 14766353 10.2741/1138

[23] Irby RB Yeatman TJ. Role of Src expression and activation in human cancer. Oncogene. (2000) 19:5636–42. 10.1038/sj.onc.1203912 11114744

[24] Summy JM Gallick GE. Src family kinases in tumor progression and metastasis. Cancer Metastasis Rev. (2003) 22:337–58. 10.1023/A:1023772912750 12884910

[25] Dehm SM Bonham K. SRC gene expression in human cancer: the role of transcriptional activation. Biochem Cell Biol. (2004) 82:263–74. 10.1139/o03-077 15060621

[26] Haura EB Tanvetyanon T Chiappori A Williams C Simon G Antonia S. Phase I/II study of the Src inhibitor dasatinib in combination with erlotinib in advanced non-small-cell lung cancer. J Clin Oncol. (2010) 28:1387–94. 10.1200/JCO.2009.25.4029 20142592 PMC3040065

[27] Johnson FM Bekele BN Feng L Wistuba I Tang XM Tran HT. Phase II study of dasatinib in patients with advanced non-small-cell lung cancer. J Clin Oncol. (2010) 28:4609–15. 10.1200/JCO.2010.30.5474 20855820 PMC2974341

[28] Johnson ML Riely GJ Rizvi NA Azzoli CG Kris MG Sima CS Phase II trial of dasatinib for patients with acquired resistance to treatment with the epidermal growth factor receptor tyrosine kinase inhibitors erlotinib or gefitinib. J Thorac Oncol. (2011) 6:1128–31. 10.1097/JTO.0b013e3182161508 21623279 PMC3230574

[29] Laurie SA Goss GD Shepherd FA Reaume MN Nicholas G Philip L. A phase II trial of saracatinib, an inhibitor of src kinases, in previously-treated advanced non-small-cell lung cancer: the princess margaret hospital phase II consortium. Clin Lung Cancer. (2014) 15:52–7. 10.1016/j.cllc.2013.08.001 24169259

[30] Boschelli DH Ye F Wang YD Dutia M Johnson SL Wu B. Optimization of 4-phenylamino-3-quinolinecarbonitriles as potent inhibitors of Src kinase activity. J Med Chem. (2001) 44:3965–77. 10.1021/jm0102250 11689083

[31] Golas JM Arndt K Etienne C Lucas J Nardin D Gibbons J. SKI-606, a 4-anilino-3-quinolinecarbonitrile dual inhibitor of Src and Abl kinases, is a potent antiproliferative agent against chronic myelogenous leukemia cells in culture and causes regression of K562 xenografts in nude mice. Cancer Res. (2003) 63:375–81. 12543790

[32] Tice DA Biscardi JS Nickles AL Parsons SJ. Mechanism of biological synergy between cellular Src and epidermal growth factor receptor. Proc Natl Acad Sci USA. (1999) 96:1415–20. 10.1073/pnas.96.4.1415 9990038 PMC15477

[33] Friday BB Adjei AA. Advances in targeting the Ras/Raf/MEK/Erk mitogen-activated protein kinase cascade with MEK inhibitors for cancer therapy. Clin Cancer Res. (2008) 14:342–6. 10.1158/1078-0432.CCR-07-4790 18223206

[34] Gilmartin AG Bleam MR Groy A Moss KG Minthorn EA Kulkarni SG. GSK1120212 (JTP-74057) is an inhibitor of MEK activity and activation with favorable pharmacokinetic properties for sustained *in vivo* pathway inhibition. Clin Cancer Res. (2011) 17:989–1000. 10.1158/1078-0432.CCR-10-2200 21245089

[35] Yamaguchi T Kakefuda R Tajima N Sowa Y Sakai T. Antitumor activities of JTP-74057 (GSK1120212), a novel MEK1/2 inhibitor, on colorectal cancer cell lines *in vitro* and *in vivo*. Int J Oncol. (2011) 39:23–31. 10.1158/1538-7445.AM2011-3585 21523318

[36] Flaherty KT Infante JR Daud A Gonzalez R Kefford RF Sosman J. Combined BRAF and MEK inhibition in melanoma with BRAF V600 mutations. N Engl J Med. (2012) 367:1694–703. 10.1056/NEJMoa1210093 23020132 PMC3549295

[37] Flaherty KT Robert C Hersey P Nathan P Garbe C Milhem M. Improved survival with MEK inhibition in BRAF-mutated melanoma. N Engl J Med. (2012) 367:107–14. 10.1056/NEJMoa1203421 22663011

[38] Okudela K Woo T Kitamura H. KRAS gene mutations in lung cancer: particulars established and issues unresolved. Pathol Int. (2010) 60:651–60. 10.1111/j.1440-1827.2010.02580.x 20846262

[39] Riely GJ Marks J Pao W. KRAS mutations in non-small cell lung cancer. Proc Am Thorac Soc. (2009) 6:201–5. 10.1513/pats.200809-107LC 19349489

[40] Sanders HR Albitar M. Somatic mutations of signaling genes in non-small-cell lung cancer. Cancer Genet Cytogenet. (2010) 203:7–15. 10.1016/j.cancergencyto.2010.07.134 20951313

[41] Eberhard DA Johnson BE Amler LC Goddard AD Heldens SL Herbst RS. Mutations in the epidermal growth factor receptor and in KRAS are predictive and prognostic indicators in patients with non-small-cell lung cancer treated with chemotherapy alone and in combination with erlotinib. J Clin Oncol. (2005) 23:5900–9. 10.1200/JCO.2005.02.857 16043828

[42] Jackman DM Miller VA Cioffredi LA Yeap BY Janne PA Riely GJ. Impact of epidermal growth factor receptor and KRAS mutations on clinical outcomes in previously untreated non-small cell lung cancer patients: results of an online tumor registry of clinical trials. Clin Cancer Res. (2009) 15:5267–73. 10.1158/1078-0432.CCR-09-0888 19671843 PMC3219530

[43] Johnson ML Sima CS Chaft J Paik PK Pao W Kris MG. Association of KRAS and EGFR mutations with survival in patients with advanced lung adenocarcinomas. Cancer. (2013) 119:356–62. 10.1002/cncr.27730 22810899 PMC3966555

[44] Mascaux C Iannino N Martin B Paesmans M Berghmans T Dusart M. The role of RAS oncogene in survival of patients with lung cancer: a systematic review of the literature with meta-analysis. Br J Cancer. (2005) 92:131–9. 10.1038/sj.bjc.6602258 15597105 PMC2361730

[45] Massarelli E Varella-Garcia M Tang X Xavier AC Ozburn NC Liu DD. KRAS mutation is an important predictor of resistance to therapy with epidermal growth factor receptor tyrosine kinase inhibitors in non-small-cell lung cancer. Clin Cancer Res. (2007) 13:2890–6. 10.1158/1078-0432.CCR-06-3043 17504988

[46] Paik PK Johnson ML D'Angelo SP Sima CS Ang D Dogan S. Driver mutations determine survival in smokers and never-smokers with stage IIIB/IV lung adenocarcinomas. Cancer. (2012) 118:5840–7. 10.1002/cncr.27637 22605530 PMC3424296

[47] Roberts PJ Stinchcombe TE Der CJ Socinski MA. Personalized medicine in non-small-cell lung cancer: is KRAS a useful marker in selecting patients for epidermal growth factor receptor-targeted therapy? J Clin Oncol. (2010) 28:4769–77. 10.1200/JCO.2009.27.4365 20921461

[48] Blumenschein GR Jr Smit EF Planchard D Kim DW Cadranel J De Pas T. A randomized phase II study of the MEK1/MEK2 inhibitor trametinib (GSK1120212) compared with docetaxel in KRAS-mutant advanced non-small-cell lung cancer (NSCLC) dagger. Ann Oncol. (2015) 26:894–901. 10.1093/annonc/mdv072 25722381 PMC4855243

[49] Jeanson A Boyer A Greillier L Tomasini P Barlesi F. Therapeutic potential of trametinib to inhibit the mutagenesis by inactivating the protein kinase pathway in non-small cell lung cancer. Expert Rev Anticancer Ther. (2018) 1–7. 10.1080/14737140.2019.1554440. [Epub ahead of print] 30513023

[50] Chalmers A Cannon L Akerley W. Adverse event management in patients with BRAF V600E-mutant non-small cell lung cancer treated with dabrafenib plus trametinib. Oncologist. (2018). 10.1634/theoncologist.2018-0296. [Epub ahead of print] 30598499 PMC6656467

[51] Gopal YN Deng W Woodman SE Komurov K Ram P Smith PD. Basal and treatment-induced activation of AKT mediates resistance to cell death by AZD6244 (ARRY-142886) in Braf-mutant human cutaneous melanoma cells. Cancer Res. (2010) 70:8736–47. 10.1158/0008-5472.CAN-10-0902 20959481 PMC4286702

[52] Yang JY Chang CJ Xia W Wang Y Wong KK Engelman JA. Activation of FOXO3a is sufficient to reverse mitogen-activated protein/extracellular signal-regulated kinase kinase inhibitor chemoresistance in human cancer. Cancer Res. (2010) 70:4709–18. 10.1158/0008-5472.CAN-09-4524 20484037 PMC2895805

[53] Nguyen KS Kobayashi S Costa DB. Acquired resistance to epidermal growth factor receptor tyrosine kinase inhibitors in non-small-cell lung cancers dependent on the epidermal growth factor receptor pathway. Clin Lung Cancer. (2009) 10:281–9. 10.3816/CLC.2009.n.039 19632948 PMC2758558

