# Supplementary figures and images for: SRC and MEK Co-inhibition Synergistically Enhances the Anti-tumor Effect in Both Non-small-cell Lung Cancer (NSCLC) and Erlotinib-Resistant NSCLC
Source: Front Oncol. 2019 Jul 30;9:586. doi: 10.3389/fonc.2019.00586 (PMC6689998; doi:10.3389/fonc.2019.00586)

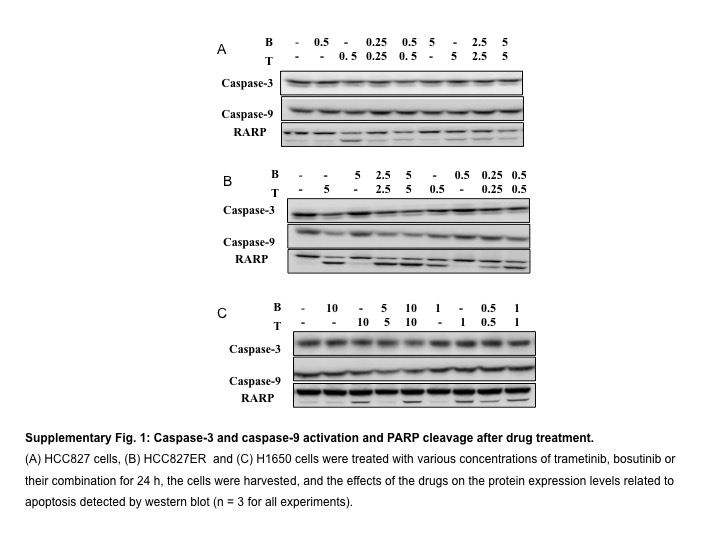

Supplement: Supplementary file 1 [file Image_1.JPEG]

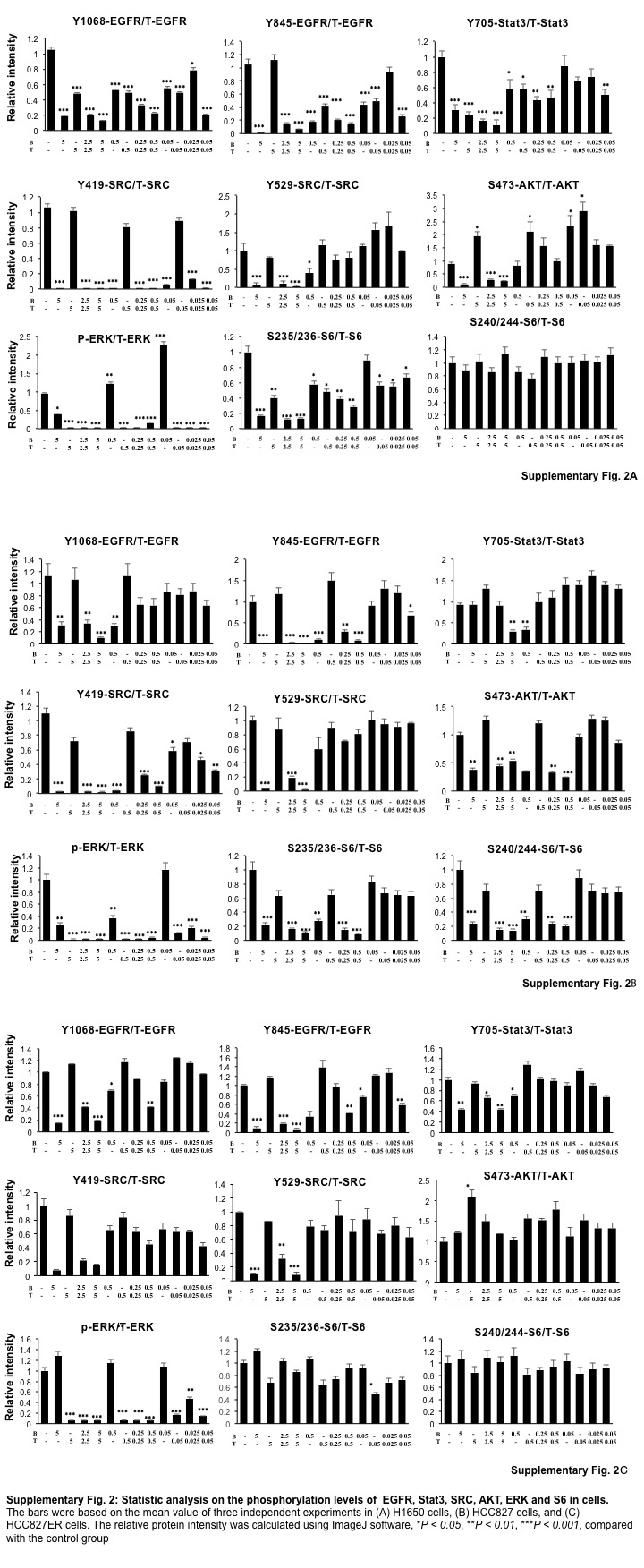

Supplement: Supplementary file 2 [file Image_2.jpg]

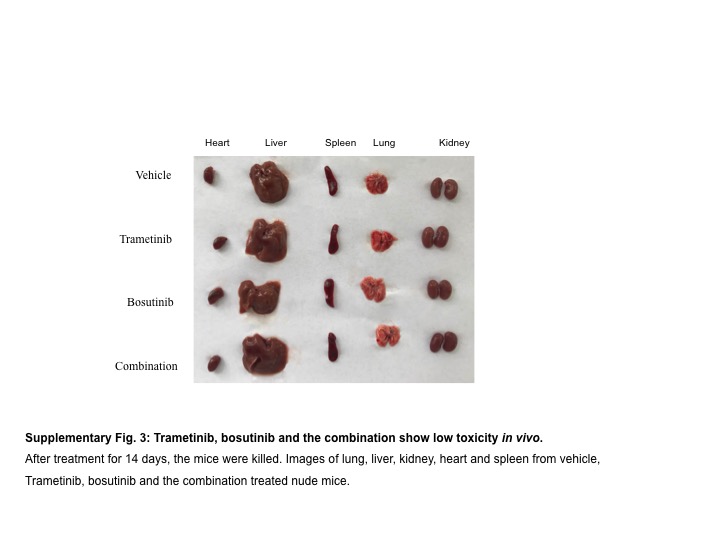

Supplement: Supplementary file 3 [file Image_3.jpeg]

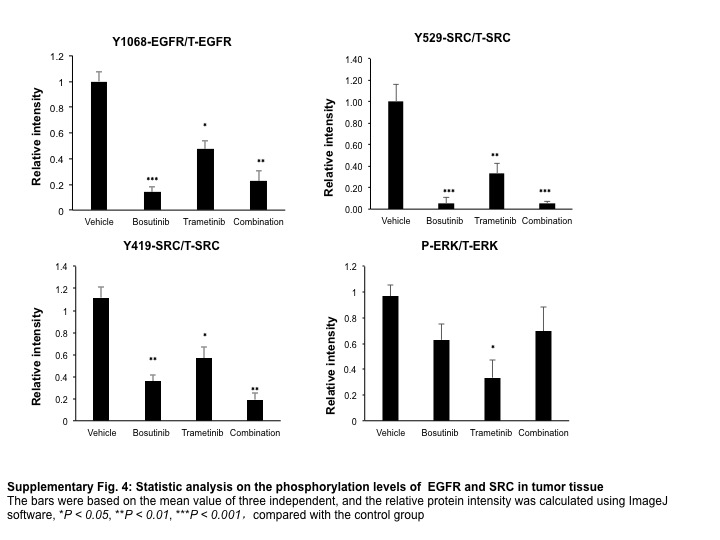

Supplement: Supplementary file 4 [file Image_4.jpeg]
